# Single-center experience with ultra-early cranioplasty within 3 weeks after decompressive craniectomy

**DOI:** 10.3389/fneur.2025.1506806

**Published:** 2025-01-20

**Authors:** Lei Zhao, Gengshen Zhang, Xiaomeng Liu, Lijun Yang, Kai Tang, Jianliang Wu

**Affiliations:** Department of Neurosurgery, Second Hospital of Hebei Medical University, Shijiazhuang, Hebei, China

**Keywords:** decompression craniotomy, ultra-early cranioplasty, surgery-related complications, neurological prognosis, duration time of surgery

## Abstract

**Background:**

The optimal timing of cranioplasty (CP) after decompressive craniectomy (DC) is inconclusive. This article aims to investigate the effect of different timing of CP on the neurologic prognosis of patients, and to explore the feasibility and safety of ultra-early CP (within 3 weeks) following DC.

**Methods:**

The duration time of surgery, intraoperative bleeding volume, surgery-related complications, and activities of daily living (ADL) scores were retrospectively analyzed in 23 patients underwent ultra-early CP performed within 3 weeks, and compared with 136 patients with non-ultra-early CP performed within the same time period.

**Results:**

The mean duration time of surgery in the ultra-early group was significantly shorter than that in the non-ultra-early group. ADL scores were significantly lower in the ultra-early group than in the non-ultra-early group both before and 1 month after CP, but there was no statistically significant difference in ADL scores between the two groups at long-term follow-up. The overall incidence of surgery-related complications was 17.39% (4/23) in the ultra-early group and 14.71% (20/136) in the non-ultra-early group, and there was no statistically significant difference in the comparison between the two groups (*p* = 0.739).

**Conclusion:**

Both ultra-early and non-ultra-early CP can significantly improve the neurological prognosis of patients. Ultra-early CP can significantly shorten the length of surgery and does not increase the incidence of surgery-related complications, which has a certain degree of safety and feasibility, and can be popularized under the premise of strict screening of indications, but further research is still needed.

## 1 Introduction

Cranioplasty (CP), which can restore a patient’s cranial appearance and structural integrity of the skull, has been described as a procedure to reduce the number of complications associated with decompression surgery and to promote neurological recovery. The optimal timing for CP is still controversial, and it is generally considered safer to perform CP 3 to 6 months after decompressive craniectomy (DC). However, increasing evidence suggests that early CP is more beneficial to the recovery of cranial nerve function (1). We have found in our clinical surgical practice that cranial repair surgery performed within 3 weeks after surgery not only makes it easier to separate the flap during surgery and causes less damage to the brain tissue, but also does not increase the complication rate.

In order to further explore the safety of performing cranial repairs within 3 weeks postoperatively, 159 patients who underwent CP in our department from 2017.8 to 2022.12 were enrolled and analyzed retrospectively. Of these, 23 patients underwent ultra-early CP (≤ 3 weeks) and 136 patients underwent non-ultra-early CP (> 3 weeks). The activities of daily living (ADL) scores were used to evaluate the patient’s neurological recovery. ADL can be used to evaluate the functional status before and after treatment and can also predict the treatment effect, length of stay and prognosis. Long-term follow-up of patients in the two groups showed no significant difference in the incidence of surgery-related complications and ADL scores between the two groups, while the duration time of operation in the ultra-early group was significantly less than that in the conventional group.

## 2 Materials and methods

### 2.1 Case selection

The following inclusion criteria were strictly followed in our group of 23 patients with ultra-early CP. Patients with cranial defects who did not fulfil the inclusion criteria underwent conventional cranial repair surgery.

Inclusion criteria: (1) The intracranial pressure was not high, and cerebral edema was completely or roughly subsided, with no clear encephalocele. (2) The flap healed well without local skin or intracranial infection. (3) The vital signs were stable. (4) There were no other serious life-threatening complications.

### 2.2 Subjects

During the period of 2017.8 to 2022.12, our department performed ultra-early CP after DC for 23 patients with skull defects who met the inclusion criteria. The baseline clinical data are shown in Table 1. The follow-up time ranged from 4 months to 53 months, and the mean follow-up time was (27.21 ± 14.44) months. Duration time of surgery, intraoperative bleeding volume and ADL score were collected from the electronic medical record system and obtained directly from the hospital big data system.

**Table 1:** Baseline clinical data of 159 patients with CP.

	Ultra-early (*n* = 23)	Non-ultra-early (*n* = 136)	*p* value
Age (yr)	44.00 ± 13.69	45.16 ± 12.56	0.816
Gender (M/F)	14/9	94/42	
Time to CP after DC (d)	17.22 ± 2.80	145.16 ± 136.32	
Primary disease			
Hypertensive intracerebral hemorrhage	7 (30.43%)	53 (38.97%)	
Cerebral hemorrhage due to AVM	1 (4.35%)	5 (3.68%)	
Severe craniocerebral injury	8 (34.78%)	62 (45.59%)	
Massive cerebral infarction	1 (4.35%)	9 (6.62%)	
Intracranial aneurysm	1 (4.35%)	7 (5.15%)	
Duration time of surgery (min)	112.78 ± 13.66	146.19 ± 20.02	0.000
Intraoperative bleeding volume (ml)	70.00 ± 33.98	87.39 ± 88.87	0.098
Subcutaneous/subdural effusion before CP	6 (26.09%)	0 (0.00%)	0.000
Complication			
Epilepsy	2 (8.70%)	13 (9.56%)	0.896
Intracranial hematoma	0 (0.00%)	2 (1.47%)	0.558
Surgical associated infection	1 (4.35%)	0 (0.00%)	0.015
Hydrocephalus and subcutaneous effusion	1 (4.35%)	5 (3.68%)	0.876
Total	4 (17.39%)	20 (14.71%)	0.739

### 2.3 Cranial replacement material selection

There are many different materials for cranial bone repair, and the most widely used include titanium mesh, polyetheretherketone and autogenous bone. Each material has its own advantages and disadvantages (2). All of these materials have been used in our center during the same period of time, but titanium mesh is still the most widely used material for cranial bone repair. In order to ensure the consistency and coherence of the experimental data, 3-dimensional (3D) plasticised titanium mesh was used as the cranial substitution material in this group of ultra-early CP patients.136 patients with non-ultra-early CP using titanium mesh as a cranial substitution material during the concurrent period were selected as controls.

### 2.4 Surgical methods

**Figure 1:**
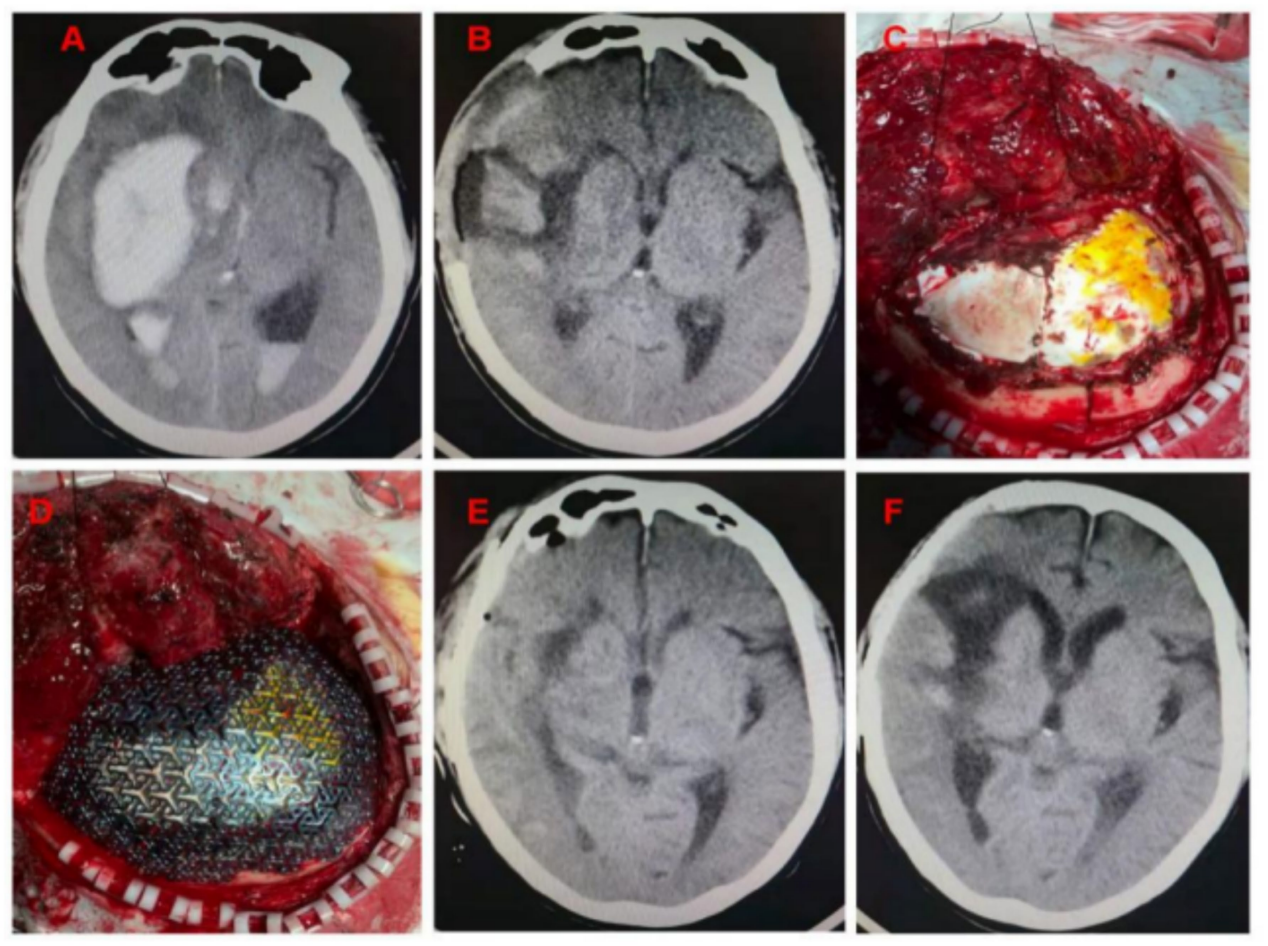
**(A)** Cerebral hemorrhage in the right basal ganglia region, cerebral hernia, midline displacement approximately 1.6 cm; **(B)** Seventeen days after DC, the patient’s brain edema decreased, and intracranial pressure was not high; **(C)** The artificial dura was intact when the flap was turned over. No pseudomembrane was observed. **(D)** The three-dimensional plastic titanium plate fit well to the skull edge. **(E)** Head CT examination on the 2nd day after surgery; **(F)** Head CT was reviewed 1 month after surgery.

All patients were treated with endotracheal intubation and general anesthesia, and the original surgical incision of DC was used. Preoperative head CT scan and skull 3D reconstruction were performed for each patient. All patients underwent CP using 3D pre-shaped titanium mesh. The amount of intraoperative bleeding volume was estimated by anesthesiologists and visiting nurses according to the suction storage device, and the duration time of surgery was timed according to the anesthesia sheet. All patients were left with a subcutaneous drain with the other end connected to a negative pressure drainage device. The removal time of the head drainage tube was determined by the drainage condition, and it was generally removed within 24–48 h after surgery. Clinical images from a representative example case are shown in Figure 1. This is a patient with a cerebral haemorrhage in the right basal ganglia region. At the time of admission, the patient already had cerebral herniation (double unequal pupils, right/left ≈5.0/2.5 mm). We performed an emergency cranial hematoma removal and decompressive craniectomy. The patient came to the hospital for re-examination 1 month after surgery, and the ADL score was 45, without epilepsy, subcutaneous effusion, or hydrocephalus.

### 2.5 Statistical analysis

Statistical analyses were conducted using SPSS Statistics (Version 26, IBM Corp.). Independent-samples *T* test and ANOVA tests were performed to compare means of continuous variables. Univariate analyses were conducted for all variables using Pearson’s chi-squared test or Fisher’s exact test for categorical variables. A *p* value <0.05 was considered statistically significant.

## 3 Results

### 3.1 Duration time of surgery was significantly shorter in the ultra-early CP group

**Figure 2:**
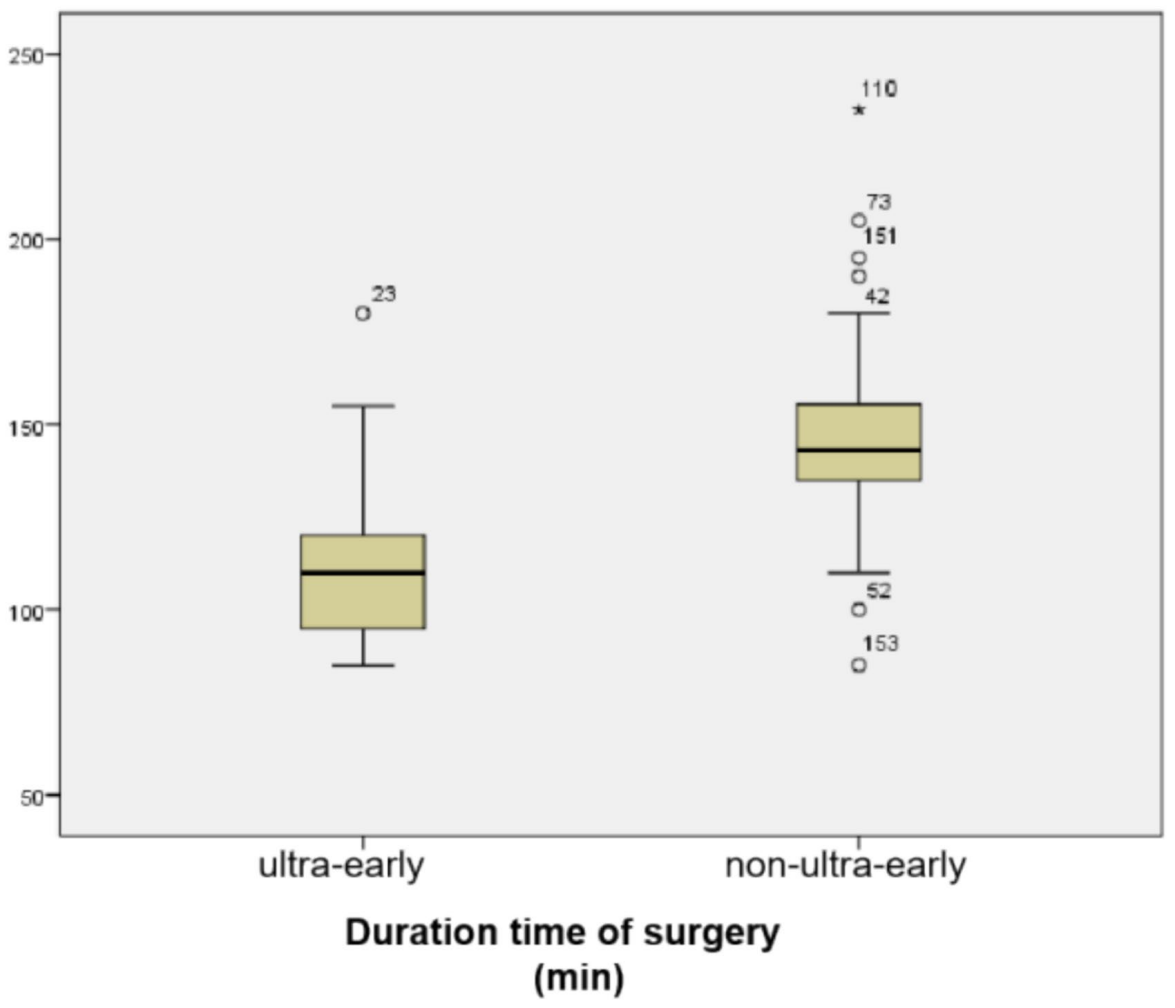
Duration time of surgery was significantly shorter in the ultra-early CP group.

The mean operative time in the ultra-early group was (112.39 ± 22.71) min, and in the non-ultra-early group was (146.19 ± 20.02) min. When comparing between the two groups, the operative time required in the ultra-early group was significantly shorter than that in the conventional group; there was a statistical difference (*p* = 0.000) (Table 1; Figure 2).

### 3.2 There was no significant difference in intraoperative bleeding volume

**Figure 3:**
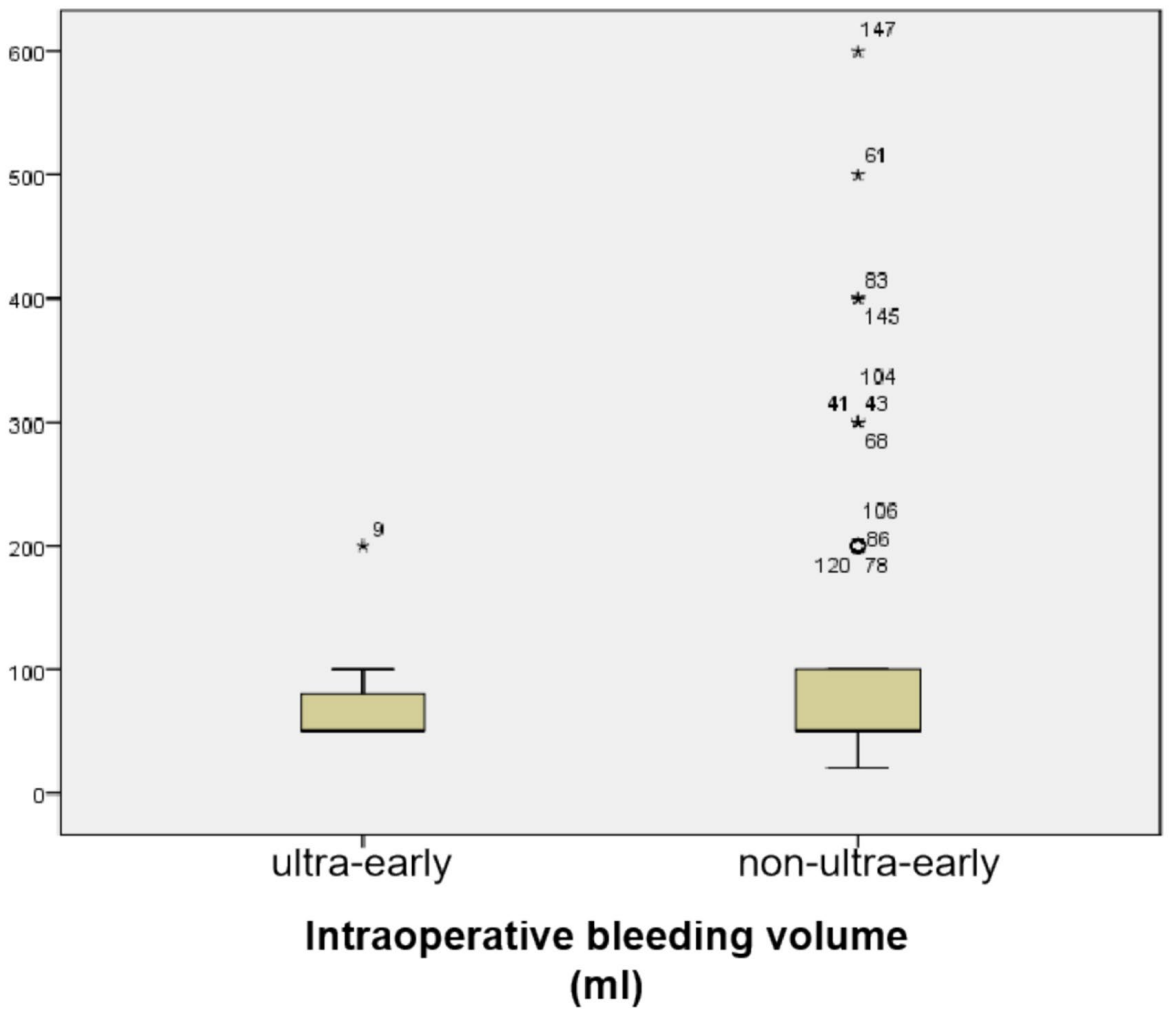
There was no statistically significant difference in the comparison between the two groups (*p* = 0.519).

The mean bleeding volume in the ultra-early group was (70.00 ± 33.98) ml, and in the non-ultra-early group it was (87.39 ± 88.87) ml; there was no statistically significant difference in the comparison between the two groups (*p* = 0.519) (Table 1; Figure 3).

### 3.3 There was no significant difference in ADL scores between the two groups of patients at long-term postoperative follow-up

**Figure 4:**
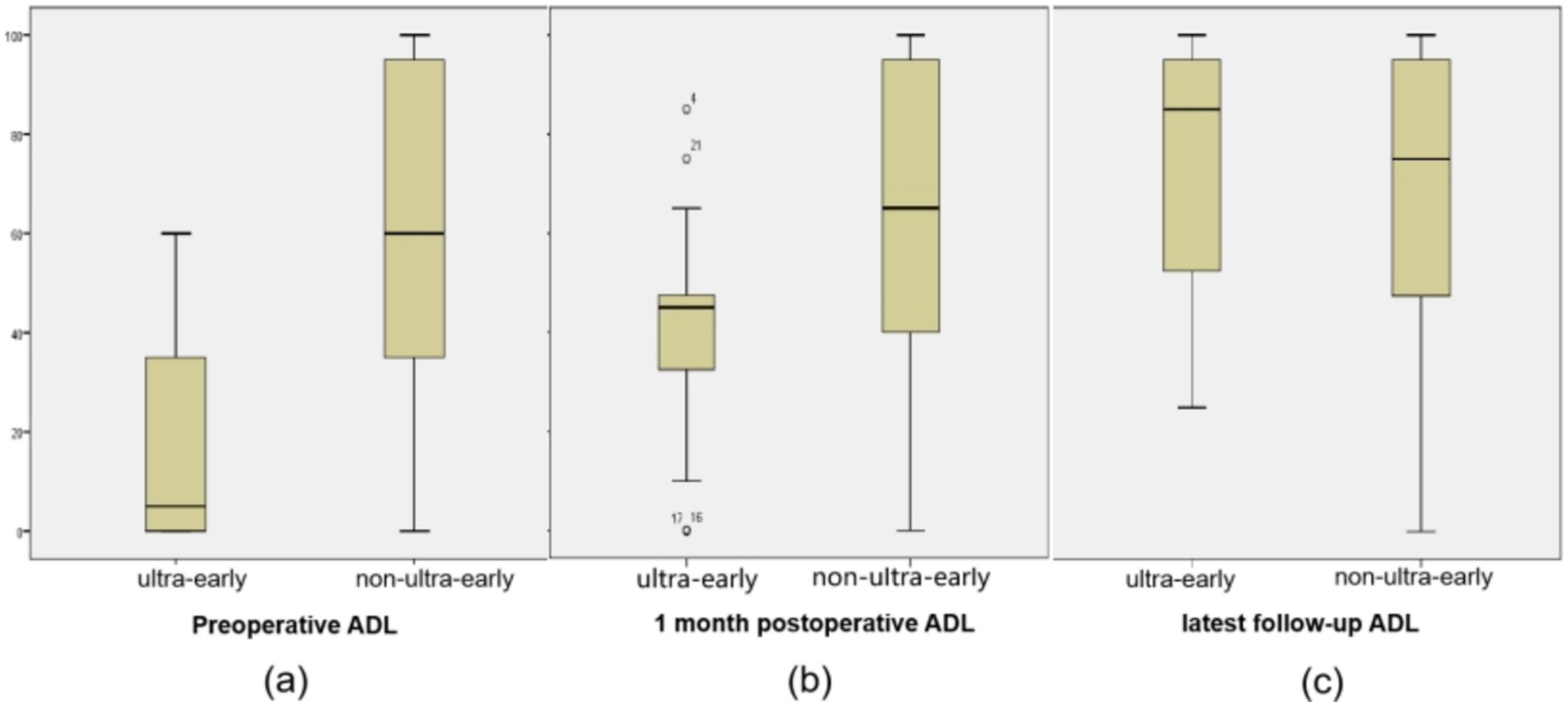
**(A)** The preoperative ADL score (17.17 ± 20.55) in the ultra-early group were significantly lower than those in the non-ultra-early group. **(B)** 1 month postoperative ADL score (41.3 ± 22.27) in the ultra-early group were significantly lower than those in the non-ultra-early group. **(C)** There was no significant difference in ADL scores between the two groups of patients at long-term postoperative follow-up.

The preoperative ADL score (17.17 ± 20.55) and 1 month postoperative ADL score (41.3 ± 22.27) in the ultra-early group were significantly lower than those in the conventional group. As for the ADL scores in the long-term postoperative follow-up, there was no significant difference between the ultra-early group (73.7 ± 24.78) and the conventional group (69.93 ± 28.76) (Table 2; Figure 4).

**Table 2:** Comparison of preoperative and postoperative ADL* scores.

	Ultra-early (*n* = 23)	Non-ultra-early (*n* = 136)	*t* value	*p* value
Preoperative ADL	17.17 ± 20.55	59.63 ± 33.00	−8.268	0.000
1 month postoperative ADL	41.30 ± 22.27	61.80 ± 31.99	−3.800	0.000
Latest follow-up ADL	73.70 ± 24.78	69.93 ± 28.76	0.592	0.555

*ADL: Assessment of daily living activities. Barthel index (BI) was used in this study. BI can be used to evaluate the functional status before and after treatment and can also predict the treatment effect, length of stay and prognosis. The Barthel index consists of 10 items and is divided into 4 functional grades of 0, 5, 10 and 15 according to the need for help and the degree of help, with a total score of 100. The higher the score, the stronger the independence and the less dependence. If the patient did not meet the criteria specified in the program, 0 points were given. A score above 60 indicates that patients can take care of themselves, 60–40 needs help, 40–20 needs much help, and below 20 needs complete help. Independent sample *T* tests were used to compare the ADL scores of patients preoperatively and postoperatively, and *p* < 0.05 indicated a significant difference.

### 3.4 There was no significant difference in the incidence of complications associated with CP

The incidence of subcutaneous effusion after ultra-early cranial repair was 4.35% (1/23), the incidence of poor scalp healing (localized infection, titanium plate removal) was 4.35% (1/23), the incidence of epilepsy after surgery was 8.70% (2/23), and the incidence of intracranial hematoma after surgery was 0% (0/23). The incidence of postoperative subcutaneous effusion in the non-ultra-early group was 3.68% (5/136), the incidence of poor scalp healing in the postoperative period was 0.00% (0/136), the incidence of postoperative seizures was 9.56% (13/136), and the incidence of postoperative intracranial hematomas (1 intracranial epidural hematoma and 1 parenchymal cerebral hemorrhage each) was 1.47% (2/136) (Table 1).

## 4 Discussion

Patients with severe craniocerebral injury, cerebral hemorrhage and other diseases accompanied by cerebral hernia often need craniotomy to remove bone flaps to maintain life and to quickly relieve intracranial pressure. However, after cerebral edema subsides and the intracranial pressure decreases, destruction of the structural integrity of the skull may lead to abnormal perfusion of local brain tissue and impaired cerebrospinal fluid (CSF) circulation. These abnormalities may lead to complications, including epilepsy, subdural effusion, and hydrocephalus (3–5). Individuals may even experience life-threatening situations such as brain tissue expansion and embedded hemorrhage due to choking and other transient increases in intracranial pressure.

CP is a routine procedure in neurosurgery. CP can repair skull defects and restore the patient’s skull appearance, structural integrity and physical protection function to reduce the occurrence of skull defect-related complications. CP can also effectively restore normal CSF dynamics and cerebral cortical blood perfusion, contributing to the recovery of patients’ neurological function (1, 6, 7). There is growing evidence that early CP significantly improves neurological outcomes (8–11). The neurologic prognostic scores (ADL scores) were significantly higher than the preoperative ones in both groups of patients in this experiment at 1 month postoperatively and at long-term follow-up. Although the 1-month postoperative ADL scores were lower in the ultra-early group than in the non-ultra-early group. However, there was no significant difference in ADL scores at long-term follow-up between the two groups. This suggests that patients are in a period of rapid neurologic recovery in the ultra-early period after DC. Furthermore, it also shows that both ultra-early and non-ultra-early cranial repairs lead to better neurological rehabilitation. Unfortunately, however, the data from examinations related to cerebral perfusion in these patients are not perfect. Therefore, we were unable to determine whether there was any difference in the improvement of cerebral perfusion between the two groups of patients.

For many years, there have been no conclusions on the appropriate timing of CP after DC surgery. Traditionally, CP is considered safer and more reliable 3 to 6 months after DC. At this time, the patient’s brain edema has subsided, and the general condition is stable (7, 12). A consensus statement was recently produced at the International Conference on Recent Advances in Neurotraumatology. The statement suggests defining the timing of cranial repair surgery as follows: ultra-early, < 6 weeks or 42 days after craniectomy; early, 6 weeks to 3 months after craniectomy; intermediate, 3–6 months after craniectomy; and delayed, >6 months after craniectomy (13).

However, in our clinical practice, we found that 4 to 6 weeks after DC, the brain surface pseudomembrane was initially formed, and it was extremely weak. In the process of flap separation, the false membrane was easily damaged and difficult to repair. At this time, the difficulty of surgery and amount of blood loss increase, and the incidence of refractory subcutaneous effusion also increase. This may be related to the destruction of the CSF circulation pathway that is being re-established. On the other hand, within 3 weeks after decompression, the artificial dural repair material has not yet been absorbed, and the pseudomembrane on the brain surface has not yet been formed. When CP is performed at this time, sharp separation flaps, such as blades and unipolar electrocoagulation, are not needed during the operation. This results in less secondary damage to brain tissue, which may be associated with less blood loss, shorter surgical time, and fewer surgical complications.

Although some studies suggest that early CP is closely related to a higher incidence of complications (14). However, the overall incidence of surgery-related complications in this group was 17.39%, similar to that reported by other studies, which report a range in complication rates from 16 to 35% (15, 16). Furthermore, it has been shown that ultra-early cranial repair does not increase the rate of surgical infection (17). There was only one patient (1/23) in our group of ultra-early CP cases developed a surgical incision infection and ultimately required a second surgery to remove the repair material. The lower surgical infection rate may be related to our more rigorous selection of patients at an earlier stage.

Through treatment of this group of cases, we found that subcutaneous or subdural fluid accumulation in the skull defect area may be the signal sent by the skull defect brain to restore its intact structure. Some scholars believe that, when brain edema is reduced, intracranial high pressure subsides, and the cranial defect side of the brain tissue pressure will be relatively low. This results in changes in CSF dynamics and may be the main cause of subdural effusions (18). Before CP, the incidence of subcutaneous/subdural effusion was 26.09% (6/23) in ultra-early group of patients who underwent DC. After CP, only 1 patient still had subcutaneous hydrocephalus. In the remaining 5 cases, subcutaneous effusion disappeared, and the flap and titanium plate were completely attached. In addition to restoring the integrity of the cranial cavity, CP can also re-establish the CSF circulation pathway to some extent (19). Therefore, ultra-early CP may be attempted in patients with skull defects with subcutaneous/subdural effusions.

## 5 Conclusion

Extensive clinical and laboratory work is still needed to confirm the safety and efficacy of ultra-early CP. On the basis of previous work, we will carry out a prospective, multicenter study with a larger sample size in combination with more intuitive and validated indicators such as cerebral perfusion studies, cerebrospinal fluid dynamics to further explore the feasibility and safety of CP at an ultra-early stage (within 3 weeks) after DC. This may make it possible for many DC patients who meet the criteria of ultra-early CP to obtain more reasonable treatment and better neurological rehabilitation.

## Data Availability

The raw data supporting the conclusions of this article will be made available by the authors, without undue reservation.
